# Multiple treatment comparisons in a series of anti-malarial trials with an ordinal primary outcome and repeated treatment evaluations

**DOI:** 10.1186/1475-2875-11-147

**Published:** 2012-05-03

**Authors:** Solange Whegang Youdom, Adeline Samson, Leonardo K Basco, Jean-Christophe Thalabard

**Affiliations:** 1 Ecole Nationale Supérieure Polytechnique, Université de Yaoundé 1, B. P. 8390 Yaoundé, Cameroon; 2 Laboratoire MAP5, UMR CNRS 8145, Université Paris Descartes, Sorbonne Paris Cité, France; 3 Institut de Recherche pour le Développement (IRD), Unité Mixte de Recherche 198, Faculté de, Médecine La Timone, Université Aix-Marseille, 13385 Marseille, France; 4 Laboratoire de Recherche sur le Paludisme, Organisation de Coordination pour la lutte contre les Endémies en Afrique Centrale (OCEAC), B. P. 288, Yaoundé, Cameroon; 5 Diagnostic Center, Hôtel-Dieu, AP-HP, Paris, France

**Keywords:** *Plasmodium falciparum*, Drug resistance, Ordinal data, Mixed models, Mixed treatment comparisons, Bayesian approach

## Abstract

**Background:**

Artemisinin-based combination therapies (ACT) are widely used in African countries, including Cameroon. Between 2005 and 2007, five randomized studies comparing different treatment arms among artesunate-amodiaquine and other ACT were conducted in Cameroonian children aged two to 60 months who had uncomplicated *Plasmodium falciparum* malaria. In these studies, the categorical criterion proposed by the World Health Organization (WHO) to assess the relative effectiveness of anti-malarial drugs was repeatedly evaluated on Days 14, 21 and 28 after treatment initiation. The aim of the present study was to compare the effects of different treatments on this repeated ordinal outcome, hence using the fully available information.

**Methods:**

The quantitative synthesis was based on individual patient data. Due to the incomplete block design concerning treatment arms between different trials, a mixed treatment comparison (MTC) meta-analysis approach was adopted. The repeated ordinal outcome was modelled through a latent variable, as a proportional odds mixed model with trial, period and treatment arms as covariates. The model was further complexified to account for the variance heterogeneity, and the individual log-residual variance was modelled as a linear mixed model, as well. The effects of individual covariates at inclusion, such as parasitaemia, fever, gender and weight, were also tested. Model parameters were estimated using a Bayesian approach *via* the WinBUGS software. After selecting the best model using Deviance Information Criterion (DIC), mixed treatment comparisons were based on the estimated treatment effects.

**Results:**

Modeling the residual variance improved the model ability to adjust the data. The results showed that, compared to artesunate-amodiaquine (ASAQ), dihydroartemisinin-piperaquine (DHPP) was significantly more efficacious. Artesunate-chlorproguanil-dapsone (ASCD) was less efficacious than artesunate-sulphadoxine-pyrimethamine (ASSP), artemether-lumefantrine (AMLM) and DHPP, the difference with the latter being significant. No difference in efficacy was found between ASAQ and AMLM.

**Conclusions:**

Bayesian mixed treatment comparisons of a network of connected randomized trials with repeated measurements of the primary categorical outcome allowed to take into account both the individual- and between- studies sources of heterogeneity. The results of the present study complete the previous quantitative review based on a binary outcome at a fixed time point, suggesting that DHPP represents an alternative for the treatment of uncomplicated *P. falciparum* malaria in Cameroonian children.

## Background

Although malaria is a global disease, over 90% of the disease burden concern the populations in sub-Saharan African countries, where *Plasmodium falciparum* particularly affects both the young children and pregnant women. Eighty-five percent of the deaths concern children less than five years of age [1].

The treatment strategy recommended by the World Health Organization (WHO) is based on artemisinin-based combination therapy (ACT), which rapidly eliminates asexual parasites. Only a few cases of treatment failure due to drug resistance have been reported so far for these treatments [2]. Artemisinin, a natural product extracted from *Artemisia annua*, was identified as a highly active anti-malarial drug. Its derivatives, such as artesunate (AS), artemether (AM) and dihydroartemisinin (DH), were found to be among the most potent of all anti-malarial drugs.

To compare the drug efficacy in Cameroon (Central Africa), a series of five trials with different combination therapies was jointly conducted by Organisation de Coordination pour la lutte contre les Endémies en Afrique Centrale (OCEAC) and Institut de Recherche pour le Développement (IRD) in Yaounde, Cameroon. The standardized 2003 WHO protocol [3] was used to assess drug efficacy in children under five years of age with a patient follow-up on days 1, 2, 3, 7, 14, 21 and 28 and evaluation of treatment outcome on day 14, day 21 and day 28. A categorical outcome with four categories is recommended in the WHO standardized protocol, which can be considered as an ordinal variable [4]: ACPR (*adequate clinical and parasitological response*), LPF (*late parasitological failure*), LCF (*late clinical failure*) and ETF (*early treatment failure*).

According to the WHO protocol for intense transmission area, ETF is defined either as a development of danger signs or severe malaria on day 1, day 2 or day 3, in the presence of parasitaemia, or parasitaemia on day 2 higher than day 0 count irrespective of axillary temperature or parasitaemia on day 3 with axillary temperature ≥ 37.5 °C or parasitaemia on day 3 ≥ 25 % of count on day 0. LCF is defined either as the development of danger signs or severe malaria after day 3 in the presence of parasitaemia, without meeting any of the previous criteria of *Early Treatment Failure* or the presence of parasitaemia and axillary temperature ≥ 37.5 °C on any day from day 4 to day 28, without meeting any of the previous criteria of *Early Treatment Failure*. LPF is defined as the presence on day 14 and after of parasitaemia and axillary temperature < 37.5 °C, without meeting any of the previous criteria of *Early Treatment Failure* and *Late Clinical Failure*. ACPR was defined as an absence of parasitaemia and axillary temperature < 37.5 °C without meeting any of the criteria of *Early Treatment Failure* or *Late Clinical Failure* or *Late Parasitological Failure*.

Each trial was conducted to test a new combination of treatments in terms of failure or success. The outcome of these studies presented a clinical heterogeneity due to the diversity of treatments and different time-periods between 2005 and 2007, which may correspond to different malaria incidences in relation to environment.

Data were first analysed with the WHO criterion considered as a binary criterion (success/failure) fitted separately on either day 14, or day 28 [5], according to the study period. A multi-treatment binary mixed-effect regression model was used with a study random effect modeling this heterogeneity. In this first analysis [5], none of the tested treatment was significantly different from the artesunate-amodiaquine (ASAQ) treatment reference arm. However, this previous work faced two limits: (i) a binary criterion was used instead of the recommended primary categorical outcome, and (ii) data were fitted separately on days 14 and 28, even though repeated measurements for each individual were available. A possible solution of the first limitation, concerning the use of a binary criterion instead of the primary categorical outcome, was proposed in a subsequent work [6], by analysing the data set on day 14 using the primary outcome as categorical. The second issue regarding repeated measurements on days 14, 21 and 28 of the primary outcome is considered in the present work with the aim to integrate the repeated measurements of the categorical outcome in a global meta-analysis approach.

Whatever the primary outcome is, the meta-analysis approach is not straightforward when more than two treatment arms are present. Indeed, a classical meta-analysis assumes identical two treatment arms in all the randomized trials. However, more complex situations occur when pooling studies with either more than two treatment arms or no identical treatment arms between studies. This results in a situation where a common treatment effect among trials cannot be easily estimated. Mixed treatment comparison (MTC) meta-analysis has been recently proposed as an extension of classical meta-analysis, by including multiple different pairwise comparisons across a range of different interventions [7]. MTC was first introduced in case of evidence synthesis [8], and is now developed in meta-analysis of clinical trials [9,10]. In this situation, MTC meta-analysis appears as a method for inferring relative treatment effects based on a synthesis of both direct and indirect evidence [11-13]. These direct and indirect evidences are usually estimated using Bayesian methods [10,14,15].

The main objective of this work was to compare the efficacy of the available combinations over time and to study the effect of individual covariates, by taking into account the subject variability and between-trials heterogeneity due to different treatment arms. A mixed model for repeated ordinal data is proposed to analyse the clinical and parasitological data. This seems to be the first mixed meta-analysis approach in pooling anti-malarial drug, in which the WHO criterion is considered as a repeated categorical outcome though with possible time-censoring.

## Data description

A total of 795 children aged two to 60 months were included in five clinical trials, one in 2005, three in 2006 and one in 2007. These successive trials corresponded to a prospective and systematic comparison of anti-malarial drugs initiated in 2003 by both OCEAC and IRD. The project at that time was approved by the Ministry of Public Health and the Cameroonian national ethics comittee. All parents or legal guardians gave their written informed consent. The data set was made available by the principal investigator of the project (LB), who initiated the present re-analysis. Data were collected based on the 2003 WHO protocol [3,16], with an evaluation at three time-points, day 14, day 21 and day 28 after the start of treatment. The primary outcome was the four categorical response, ACPR, LPF, LCF, ETF, which was repeatedly recorded on days 14, 21 and 28. The category ETF was, in fact, never observed. Individual covariates such as weight, age, gender, parasitaemia on day 0 and day 3 were also measured. Each trial compared two or three treatments among either amodiaquine (AQ) in monotherapy or a combined treatment such as amodiaquine-sulphadoxine-pyrimethamine (AQSP), artesunate-amodiaquine (ASAQ), artesunate-sulpha doxine-pyrimethamine (ASSP), artesunate-mefloquine (ASMQ), artemether-lumefantrine (AMLM), dihydroartemisinin-piperaquine (DHPP).

**Figure 1:**
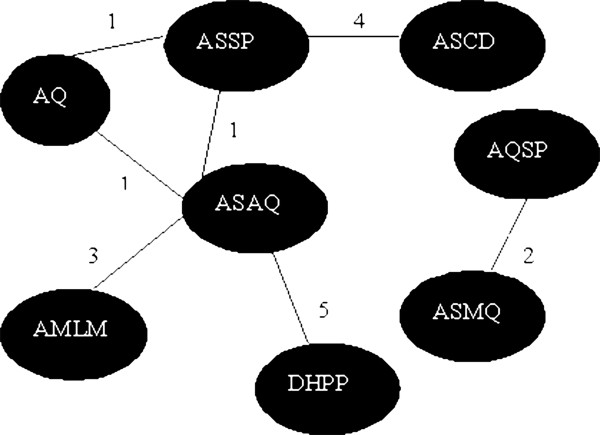
**Treatment arms of the five clinical trials.** Each edge is attributed two subscripts, the first one identifies the randomized clinical trial, the second one identifies the possibility of a direct comparison between two nodes within a randomized clinical trial. Only the study #2, which compared AQSP to ASMQ, is not connected to the others.

Figure 1 represents the set of treatment arms as a graph, in which each treatment arm is a node. An edge between two nodes exists when they were tested in the same randomized clinical subtrial. This graph can be viewed as a connected network with the exception of study # 2, which is unconnected to the other studies. As suggested by Jansen *et al.*[10], the following analysis was conducted without this specific study since no increase in the corresponding treatment effect estimates could be gained by pooling this specific trial with the others.

The sample size for each treatment arm was initially set at 50 children, then adjusted to account for drop-outs. Details of the observed categorical outcomes, including missing responses (NA) according to each treatment arm within the different trials are shown in Table 1. Forty-nine children (6.1%) were either excluded or lost to follow up before day 14. Consequently, 746 patients had their responses assessed on day 14. Missing responses on day 21 and day 28 were related to previously observed treatment failure or lost to follow-up after day 14. Indeed, as soon as a failure was noticed, the patients received an alternative drug to prevent clinical aggravation and progression towards potentially severe and complicated malaria. Finally, after excluding patients from study # 2, 621 patients were analysed in a per-protocol approach (PP). The sensitivity of the models to the missing responses was assessed by imputing the missing responses after day 14 using different scenarii, including the less favorable scenario in the course of the treated acute episode.

**Table 1:** Summary of the patient populations by treatment arm in the five clinical trials

						**Day14**			**Day21**				**Day28**			
**Study**	**Year**	**Treat**	***m***_**1**_	***m***_**2**_	***m***_**3**_	**ACPR**	**LPF**	**LCF**	**ACPR**	**LPF**	**LCF**	**NA**	**ACPR**	**LPF**	**LCF**	**NA**
#																
	2005	AQ	64	3	61	58	2	1	54	0	2	5	50	2	2	7
	*Feb-May*	ASAQ	60	3	57	56	0	1	49	0	4	4	43	3	3	8
1		ASSP	61	3	58	58	0	0	55	1	1	1	50	3	2	3
	Subtotal		**185**	9	**176**	172	2	2	158	1	7	10	143	8	7	**18**
	2006 1	AQSP	67	3	64	64	0	0	61	1	1	1	55	1	4	4
	*Apr-Jul*	ASMQ	69	8	61	61	0	0	61	0	0	0	60	1	0	0
2	Subtotal		**136**	11	**125**	125	0	0	122	1	1	1	115	2	4	**4**
	2006 2	ASAQ	62	4	58	58	0	0	52	4	1	1	52	0	0	6
	*Sep-Nov*	AMLM	61	1	60	60	0	0	58	2	0	0	58	0	0	2
3	Subtotal		**123**	5	**118**	118	0	0	110	6	1	1	110	0	0	**8**
	2006 3	ASCD	86	14	72	71	1	0	57	6	8	1	53	2	1	16
4	*Dec-Feb*	ASSP	82	1	81	81	0	0	79	0	2	0	74	2	2	3
	Subtotal		**168**	15	**153**	152	1	0	136	6	10	1	110	0	0	**19**
	2007	ASAQ	92	4	88	87	1	0	78	6	3	1	73	5	0	10
5	*Apr-Jul*	DHPP	91	5	86	86	0	0	86	0	0	0	84	2	0	0
	Subtotal		**183**	9	**164**	173	1	0	164	6	3	1	157	7	0	**10**
	Subtotal	*ASAQ*	214	11	203	201	1	1	179	10	8	6	168	8	3	24
		Others	581	38	543	539	3	1	513	10	14	8	484	13	11	35
	Total		**795**	*49*	**746**	740	4	2	690	20	22	14	652	21	14	**59**

*m*_1_: number of included patients, *m*_2_: number of drop-out and lost to follow-up before Day 14, *m*_3_: number of evaluated patients on Day 14. ACPR *adequate clinical and parasitological response*, LPF *late parasitological failure*, LCF *late clinical failure*), NA: not available outcome. AQ, amodiaquine; SP, sulphadoxine-pyrimethamine; AS, artesunate; AM, artemether; DHPP, dihydroartemisinin-piperaquine; LM, lumefantrine; CD, chlorproguanil-dapsone.

## Statistical analyses

The categorical outcome was regressed *via* a latent continuous variable on various covariates of interest, using either fixed- or mixed-model formulation, with an attempt to take into account the individual response heterogeneity by modeling the residual variance.

### Notations and associated latent variable modeling

The categorical ordinal outcome was measured in the studies _*Sm*_ where $m\in \left\{1,3,4,5\right\}$, as study _*S*2_ was removed for the present analysis. Each study _*Sm*_had *i*=1, … ,_*Nm*_ subjects observed at times *t*=1, … ,_*Omi*_for subject *i*. Let _*Zmit*_be the *K*=3 category response of subject *i* at time *t* in study *m* coded as 1 for ACPR, 2 for LPF and 3 for LCF, respectively, as the ETF category was never observed.

Categorical ordinal data are commonly analysed using an ordinal logistic model [17]. Briefly, this model assumes an underlying latent variable that is related to the ordinal response _*Zmit*_ through threshold values. This latent variable corresponds to an unobserved variable, measured on a continuous scale which drives, in each infected patient, the patient’s response to the anti-malarial drug, he is exposed to. This latent variable is assumed to depend on observed covariates like patient’s nutritional status, *Plasmodium* exposure, mosquito bite intensity, level of education or family income for each individual with an heterogeneity between subjects and is modelled accordingly. The thresholds correspond to different distinct values, depending on the number of ordinal categories that separate individuals into various response categories. The response of a given subject is determined by the interval in which its unobserved latent variable falls. Let _*Lmit*_ be the corresponding latent variable and _*αk*_*k*=1,…,*K*−1, the thresholds which are assumed $-\infty =\alpha_{0}<\alpha_{1}\leq \ldots \leq \alpha_{K-1}\leq \alpha_{K}=+\infty$. The relationship between between _*Zmit*_and _*Lmit*_ can be defined as follows

$$Z_{\mathrm{mit}}=k\Leftrightarrow \alpha_{k-1}\leq L_{\mathrm{mit}}<\alpha_{k}$$

Let _*Qmitk*_=*Pr*(_*Zmit*_≤*k*)=*Pr*(_*Lmit*_≤_*αk*_) be the cumulative probability. A natural statistical model links the cumulated probabilities _*Qmitk*_ to the covariates *via* a link function *g*. Assuming a normal or logistic distribution for the underlying latent variable leads to an ordinal probit regression model or an ordinal logistic regression model, respectively. The complementary log-log link function can also be used. In the following, a logit link function which corresponds to the proportional odds model is used.

The latent variable _*Lmit*_was assumed to follow a mixed logit model and could be expressed in the following way:

$$L_{\mathrm{mit}}=X_{\mathrm{mit}}\gamma +u_{i}+e_{\mathrm{mit}}$$

where _*Xmit*_ is the matrix of covariates, *γ* is the covariate parameter vector, _*ui*_is the subject random effect, assumed to follow a centered gaussian distribution with constant variance ^*τ*2^. The residuals _*emit*_, were assumed to be distributed according to a centered logistic random variable with variance _*σmit*_. Conditionally to _*ui*_, this model is equivalent to

$$\mathrm{logit}(Q_{\mathrm{mitk}})=(\alpha_{k}-X_{\mathrm{mit}}\gamma -u_{i})/\sigma_{\mathrm{mit}}.$$

The covariate model _*Xmit*_and the variances _*σmit*_are detailed in the next two sections. No study random effect was introduced as the number of studies was small.

### Covariate model

The covariate model was the following:

(1) $$X_{\mathrm{mit}}\gamma =\overset{6}{\underset{l=2}{\sum }}\gamma_{l}\mathrm{Tr}t_{\mathrm{il}}+\overset{3}{\underset{t=2}{\sum }}\gamma_{t}\mathrm{Tim}e_{\mathrm{it}}+\overset{5}{\underset{m=3}{\sum }}\gamma_{m}\mathrm{Stud}y_{\mathrm{im}},$$

where *Tr*_*til*_*l*=2,…,6, are binary treatment covariates coding for the 6 different treatments; *Tim*_*eit*_*t*=2,3, are binary covariates coding for the _*Tm*_=3 measurement times (day 14, day 21, day 28) and, *Stud*_*yim*_*m*=3,4,5 are 3 binary covariates coding for the four studies. Day 14 was selected as the reference time, study 1 as the reference study. The ASAQ treatment was the reference treatment as it is currently in use in Cameroon, as well as in many other African countries [18]. Comparison was made between ASAQ and the remaining 5 treatments $\left\{\mathrm{AQ},\mathrm{ASSP},\mathrm{ASCD},\mathrm{AMLM},\mathrm{DHPP}\right\}$.

The parameter vector, *γ*=(_*γl*_,_*γt*_,_*γm*_) represents the effect of the selected covariates, i.e., population mean, treatment, time, and study, respectively. Each _*γl*_represents the treatment effect difference between ASAQ and the corresponding *l*th treatment, assuming the other fixed.

This general model assumes: i) that the same number of categories for the outcome holds both in each study and at the different time visit; ii) that, at each visit, the treatment differential effects verify the proportional odds hypothesis between studies. Each parameter is represented in terms of the logarithm of the cumulative Odds Ratio (logOR). The corresponding OR is obtained by taking the exponential. Because data were treated as categorical, log(OR) represents the posterior cumulative logarithm of odds ratio, linking ACPR to LPF. A log(OR) greater than 0 with a 95% confidence interval not containing 0 means that there is an improvement of subject status over time for a given treatment compared to the reference treatment. A positive regression coefficient means that the effects proceed towards the best response (ACPR), whereas a negative regression coefficient means that the effects go towards the worst response (LPF here).

### Modeling the residual

The simplest residual variance model is the classical constant model (_*σmit*_=*σ*). This model is referred as Model M1 or homogeneity model in the following sections. Because of the presence of multi-treatments and heterogeneity of the discrete outcomes among treatment arms, a more flexible, yet parsimonious, general model was introduced to take into account the large heterogeneity of the residual variance. This approach is derived from Foulley & Jaffrezic [19], who proposed to model the residual variance as a function of some relevant covariates. Relevant covariates like treatment arms, different follow-up period, the different studies, and a subject random effect, were selected to enter the model. As described by Foulley & Jaffrezic[19], a structural mixed model on the log of variances was proposed:

(2) $$\;\;\;\;\;\;\;\;\log\overset{2}{\underset{\mathrm{mit}}{\sigma }}=\overset{6}{\underset{l=2}{\sum }}\delta_{l}\mathrm{Tr}t_{\mathrm{il}}+\overset{3}{\underset{t=2}{\sum }}\delta_{h}\mathrm{Tim}e_{\mathrm{it}}+\overset{5}{\underset{m=3}{\sum }}\delta_{m}\mathrm{Stud}y_{\mathrm{im}}+v_{i},$$

where $v_{i}∼풩(0,\eta^{2})$ models the between-subject heterogeneity of the discrete outcome, and the vector *δ*=(_*δl*__*δh*__*δm*_) represents the vector of covariates effects to be estimated. This leads to model M2.

Model M3 discarded the subject-specific random effect _*vi*_ on the log of the variances in equation (2):

(3) $$\log\overset{2}{\underset{\mathrm{mit}}{\sigma }}=\overset{6}{\underset{l=2}{\sum }}\delta_{l}\mathrm{Tr}t_{\mathrm{il}}+\overset{3}{\underset{t=2}{\sum }}\delta_{h}\mathrm{Tim}e_{\mathrm{it}}+\overset{5}{\underset{m=3}{\sum }}\delta_{m}\mathrm{Stud}y_{\mathrm{im}}.$$

Model M4 kept only the treatment effects in equation (2) to form the following:

(4) $$\log\overset{2}{\underset{i}{\sigma }}=\overset{6}{\underset{l=2}{\sum }}\delta_{l}\mathrm{Tr}t_{\mathrm{il}}.$$

Finally, another model called M5 was also considered by adding a subject random effect to equation (4), corresponding to:

(5) $$\log\overset{2}{\underset{i}{\sigma }}=\overset{6}{\underset{l=2}{\sum }}\delta_{l}\mathrm{Tr}t_{\mathrm{il}}+v_{i}.$$

### Parameter estimation, model comparison and validation

All analyses were based on individual patient data. A Bayesian approach under the WinBUGS software [20] was used to estimate the parameters of models M1-M5, as it handles easily hierarchical logistic models, together with model selection and model validation. The estimation procedure was based on Gibbs sampling. The above models were implemented by setting priors to all parameters. The threshold values _*αk*_ were sampled as follows: _*α*1_ was set to zero, _*α*2_ was assumed _*α*1_ + *Δ*, where *Δ* followed a uniform prior distribution 풰(0,5). Priors for the time and study effects were assumed 풰(−5,5), whereas priors for the variances ^*τ*2^and ^*η*2^ were choosen 풰(0,80) and 풰(0,4), respectively, as suggested by [19], where 풰(a,b) corresponds to a uniform distribution on the interval (*ab*). Priors for the other individual covariates in the different variance models, like weight, gender, age, parasitaemia, were assumed normally distributed 풩(0,100).

All Bayesian analyses were performed using one chain of 50,000 samples, the first 25,000 of which were removed to allow for burn-in. Credibility intervals were estimated together with the parameters. All parameters were expressed as logarithm of odds ratios. In practice, when these intervals contained zero, the parameter was considered to be not different from 0. To take into account the problem of multi-treatment comparisons, the Bonferroni correction was used. The different models were compared using the DIC (*deviance information criterion*) [21]. The best model was considered to be the one with the smallest DIC.

### Sensitivity analysis and missing responses imputation

Limiting the analysis to the observed responses only exposes to biases, as it does not take into account the responses recorded as NA, the details of which are given in Table 2. These NA responses could have several origins: i) the absence of the child at the time of the scheduled visit, due to some particular familial event related or not to the perceived health status of the child by the parents. The field worker usually managed to visit the family when they returned and record the outcome; ii) the decision to switch to another treatment before the scheduled visit when, at the previous visit or in the time interval between the two visits, the observed outcome suggested treatment failure and motivated the switch. Even if the child was still followed up in the trial design, his or her real status outcome was recorded not applicable. In the following these responses will be called missing, although they were recorded but considered not applicable, as the child was no longer under the allocated treatment. In order to test the sensitivity of the results to these missing responses, missing responses were replaced with the last observed response which was carried forward to all the following visits, except situations #1 and #2 in Table 2, which were imputed with the less favorable scenario LCF. This approach is refered as the imputation approach therafter.

**Table 2:** Number of children by outcomes at the three scheduled visits

**Type of situation**	**Day 14**	**Day 21**	**Day 28**	**Number of children (%)**
1	ACPR	NA **(LCF)**	NA **(LCF)**	8 (1.1)
2	ACPR	ACPR	NA **(LCF)**	4 (0.4)
3	ACPR	LPF	NA **(LPF)**	20 (2.5)
4	ACPR	LCF	NA **(LCF)**	21 (2.6)
5	NA	NA	NA	49 (6.2)
6	LCF	NA **(LCF)**	NA **(LCF**)	2 (0.3)
7	LPF	NA **(LPF)**	NA **(LPF)**	4 (0.4)
8	ETF	NA **(ETF)**	NA **(ETF)**	**0** (0.0)
9	ACPR	ACPR	ACPR	652 (82)
10	ACPR	ACPR	LPF	21 (2.6)
11	ACPR	ACPR	LCF	14 (1.8)
Expected children	795	746	732	
Missing	49	14	59	122 (15)
Fully observed	746	732	687	

ACPR: Adequate Clinical and Parasitological Response. LPF: Late Parasitological Failure. LCF: Late Clinical Failure. ETF: Early Treatment Failure. NA: missing outcome. The imputed states in the sensitivity analysis are indicated in brackets.

When a failure was recorded, blood samples were tested using PCR for assessing either the persistance of the infection, i.e. a real treatment failure, or a new infection with a different parasite strain. In the latter situation, treatment failure could be cancelled and imputed ACPR. This PCR- corrected data set was analysed in the sensitivity analysis to compare with the PP analysis.

## Results

Individual covariates, i.e., weight, gender, age, and parasitaemia, were tested in a fixed effect model together with treatment, time and study-effects using a fixed effect model, in the PP approach. As shown in Table 2, none of the individual covariates was significant, whereas both time- and treatment- effects were significant. ASCD appeared significantly less efficacious than ASSP. The negative time effect coefficient suggested that the complete response (ACPR) decreased significantly over time, from day 14 to day 28. This result can be related to a loss of improvement due to treatment failure caused by re-infections or recrudescence over time, or an overall efficacy of drugs as early as Day 14. The results for the other analyses performed with models M1, M2, M3, M4 and M5 without individual covariates, are displayed in Table 3 for both PP- and imputation- approaches. Both PP- and imputation- approaches gave similar results.

**Table 3:** Estimated study- treatment-, time- and individual covariate- effects using a fixed effect model

**Parameters**	**mean**	**sd**	**2.5%**	**97.5%**
_*α*1_	4.91*	0.71	3.61	6.42
_*α*2_	5.77*	0.72	4.45	7.29
_*γS*3_ (Study 3-1)	0.246	0.537	-0.742	1.371
_*γS*4_ (Study 4-1)	0.572	0.584	−0.547	1.719
_*γS*5_ (Study 5-1)	−0.505	0.367	−1.216	0.222
_*γ*2_ (AQ)	−0.402	0.434	−1.218	0.481
_*γ*3_ (AMLM)	1.212	0.936	−0.443	3.24
_*γ*4_ (ASCD)	−1.514*	0.682	−2.85	−0.186
_*γ*5_ (ASSP)	−0.109	0.465	−0.989	0.844
_*γ*6_ (DHPP)	2.353*	0.852	0.924	4.288
_*γD*21_ (Day 21–14)	−2.121*	0.459	−3.112*	−1.298
_*γD*28_ (Day 28–14)	−2.036*	0.467	−3.030*	−1.194
Weight	0.218	0.360	−0.478	0.933
Gender	−0.154	0.234	−0.615	0.298
Age	0.189	0.505	−0.896	1.067
Parasitaemia Day 0	−0.171	0.352	−0.872	0.506
Parasitaemia Day 3	0.085	0.669	−1.083	1.542
Expected children	795	746	732	
Missing	49	14	59	122 (15)
Fully observed	746	732	687	

sd: standard deviation, 2.5% and 97.5% : percentiles of the posterior distributions. Study reference: Study 1; Treatment reference: ASAQ; Day reference: Day 14. *: significant effect at the 0.05*%*level.

### Comparison and validation of the different approaches

Different models were compared, starting with the standard threshold model with homogeneous variance (M1). Modeling the heterogeneity of residuals considerably improved the fit as shown in Table 4. This suggested a gain in modeling the heterogeneity of residuals in the presence of the treatment-, time- and study- covariates. However, this gain decreased when modeling only the treatment effect within the residual variance. As shown in Table 4, the DICs in models M4 and M5 are larger than DICs in models M3 and M2. Consequently, the best model was M3 for the PP approach and M2 for the imputation approach.

**Table 4:** Estimated effects for models M1-M5 in the per-protocol (PP) analysis

	**M1**		**M2**		**M3**			**M4**			**M5**		
	**Est**	**SD**	**Est**	**SD**	**Est**	**SD**		**Est**	**SD**		**Est**	**SD**	
Study 3-1	2.47	1.01	−1.26	1.61	0.10	1.20		1.70	0.93		1.71	0.98	
Study 4-1	1.03	1.26	1.33	1.40	0.78	1.04		1.07	1.04		1.03	1.12	
Study 5-1	1.08	1.07	−1.88	1.23	−0.66	1.03		0.49	0.76		0.27	0.85	
AQ	0.68	1.23	1.35	1.39	0.39	0.92		0.35	0.86		0.45	0.97	
AMLM	2.04	1.30	1.50	1.20	1.35	1.07		1.65	1.15		1.71	1.23	
ASCD	−1.76	1.37	−1.97	1.41	−1.25	1.24		−1.56	1.22		−1.69	1.26	
ASSP	−1.77	1.10	2.31*	1.09	1.59	0.95		2.22*	1.07		2.00	1.20	
DHPP	3.09*	0.74	2.93*	0.77	2.88*	0.68		2.27*	0.94		2.04*	1.04	
_*γD*21_ (Day21-14)	−4.17*	0.60	−3.14*	1.50	−2.99*	0.72		−3.28*	0.57		−3.46	0.61	
_*γD*28_ (Day28-14)	−3.87*	0.98	−3.04*	1.53	−3.03*	1.10		−3.69*	0.60		−3.91*	0.63	
*τ*	6.57*	1.73	6.89*	1.75	7.52*	1.16		7.77*	1.00		7.40*	1.29	
*η*			0.80*	0.26							0.34	0.29	
DIC	636.69		371.24		302.32			536.82			532.07		

Study # 2, excluded. Est=estimation; SD=standard deviation; *: significant effect at the 5% level. *τ*: standard error of the subject random effect in modeling the logit- cumulated categorical variable. *η*: standard error of the subject random effect in modeling the log- variance (model 2).

### Direct and indirect comparisons

**Figure 2:**
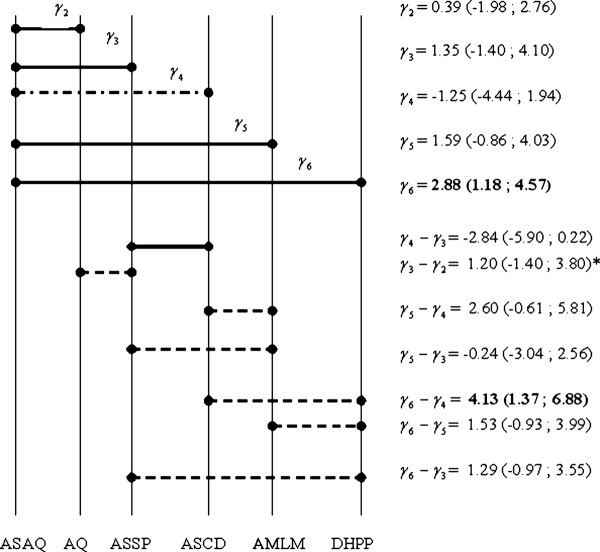
**Direct and Indirect treatment differences based on the PP approach with the M3 model (Study # 2, excluded).***γ*represents the logarithm of the cumulative *OR*. Each treatment is compared to ASAQ (_*γ*1_=0). Solid lines mean that the corresponding treatments were tested within the same study, whereas dashed lines correspond to treatments compared between different studies. Values in **bold** are significant differences. * means that a correlation is taken into account for CI (credibility interval) as AQ, ASSP and ASAQ are tested within the same study.

The model M3, which gave the best adjustment to the data set, was selected for a simultaneous comparison between treatments. Figure 2 shows the treatment effects estimated in Model M3 using the PP approach, ranked in chronological order of the trials. Direct and indirect treatment effects could then be computed and are represented in the same figure.

ASAQ and DHPP treatments differed significantly. DHPP was globally more efficacious than ASAQ (logOR=2.88, 95*%CI*=[1.18;4.57]). DHPP was more efficacious than ASCD (logOR=4.13, 95*%CI*=[1.37;6.88]). As compared to AMLM, the combination ASCD was less efficacious (logOR=−2.60, 95*%CI*=[−5.81;0.61]); ASCD was found less efficacious than ASSP (logOR=−2.84, 95*%CI*=[−5.90;0.22]), though both differences were not significant. All the other direct and indirect comparisons did not differ significantly as well.

## Discussion

The aim of this work was to pool the results from five randomized clinical trials comparing the efficacy of anti-malarial drugs based on the same repeated observation-time design but with partially overlapping treatment arms, in order to improve the estimated treatment effects and their corresponding variances. Among the five studies, one was discarded (study #2) as it was not connected to the other ones, whereas the four remaining studies (studies # 1,3,4,5) were analysed with a mixed ordinal logistic model incorporating a between-subject heterogeneity variance model. This approach can be considered as an extension of the multi-treatment approach of Jansen *et al.*[22], which was limited to a binary response at a single time point (day 28) or, an alternative to a recent work carried by Dakin *et al.*[23], where the outcome was continuous. Results concerning the unconnected study are just mentioned for the sake of completeness, as they were not part of the global analysis: they showed that the combination ASMQ was more effective than AQSP. In the global analysis, the best model was M3 with a random individual effect, in which the residual variance was a function of explanatory covariates. Modeling the subject residual variance appeared to improve the model ability to fit the data by reducing heterogeneity within the analysed trials. Based on model M3, DHPP was significantly more efficacious than ASAQ, whereas ASCD appeared less efficacious than ASSP, AMLM and DHPP, the latter difference being significant. These results slightly differ from the results of our previous work [5], in which no significant treatment difference was found. Therefore, taking into consideration both the ordinal type of the WHO criteria and the results at the repeated visits seems to increase the power for finding a difference, if any.

Regarding the categorical outcome, LCF is symptomatic, whereas LPF is not. It remains possible that a patient with LPF become symptomatic beyond day 28. However, the protocol was designed to separate the two endpoints when performing a 28-day treatment evaluation. The clinical implications of LPF and LCF on day 28 seem to be quite different. Moreover, the more recent WHO document [24] maintains the four treatment outcomes.

In the present study, analyses were based on the observed treatment responses between day 14 and day 28 (due to the absence of ETF), and the contribution of each observed category was evaluated. Pooling randomized clinical trials raises the issue of heterogeneity between studies. However, all studies included in this analysis were based on the same population of children, within the same age range and in the same geographic area. These studies had the same design and were run by the same investigators and field workers over the years. Mixed treatment comparison (MTC) meta-analysis faces several limits leading to the possibility of biased estimates. Comparing treatment arms using indirect comparisons apparently exposes to the loss of the benefits of randomization. However, it is partially preserved using adjusted comparisons with possibly less biased differences towards positive results, according to Song *et al.*[25]. A study random effect was not considered in the models as the number of studies was too small. None of the study fixed effects was significant, but including them in the model allowed for a correlation between the treatment arms within a single study, which kept part of the randomisation process. Missing responses represent a frequent issue in anti-malarials trials, usually carried out in field conditions. The absence of a patient during a scheduled visit could be due either to an earlier treatment failure leading to another treatment, which could be considered as missing at random (MAR), according to Rubin [26], or a lost to follow-up considered as missing completely at random (MCAR) or an exclusion due to some protocol violation, considered as missing not at random (MNAR). In order to explore the internal validity of our results, a sensitivity analysis was carried out in which missing responses were imputed according to different scenarii, including the worst scenario where missing responses were imputed as failures. None of the evaluations carried out before day 14 (i.e. days 1, 2, 3, and 7) was considered because early treatment failure (ETF, for days 1 to 3) and late failure between day 7 and day 13 were not observed. In addition the whole purpose for WHO to extend the follow up beyond day 14 up to day 28 was to study the long term efficacy of anti-malarial drugs following an acute episode. Each of the 3 categories ACPR, LCF, LPF on days 14, 21 and 28, according to the 2003 WHO protocol, was observed. On the observed data, one subject cannot be LPF or LCF without being ACPR at least on day 14. When the outcome of a subject is classified as LPF or LCF, the next outcome is missing since the evaluation of drug efficacy is terminated for that particular patient and an alternative treatment is necessary because of ethical consideration. Therefore, outcomes are not strictly speaking repeated. Modeling repeated observations over time can been achieved either using a conditional model, where the outcome at time t is modelled according to the previous outcomes, or using a marginal model, where the individual outcomes are modelled in relation to a mean outcome at each time- point, the time dependency reflecting this memory effect acting on the categorical response. As the main objective of the present work was to pool the results of different multi- arm trials, the latter approach was adopted, which could be directly related to recent advances in meta-analysis developments [27]. Analyses with incomplete (PP) and complete outcomes (imputation approach) were performed by imputing missing outcomes on days 14, 21 and 28. The results were then compared to check for biases (Table 5). The results remained similar in all approaches. It is now common in anti-malarial drug trials to distinguish between new infections and recrudescence by PCR, although, from the pragmatic point of view, one might expect that an optimal treatment of an acute episode would protect the patients from new infection in the weeks following the episode, in areas without a large variability in parasite phenotype. In case of unevenly distributed missing categories at different times and/or treatment arms, difficulties in adjusting the proposed models could occur. The main difficulty was related either to a too small number or an absence of failure categories over time after PCR correction. This could be considered as extreme category outcomes, for which the clog- log link can be more adapted than the logit link in the fitting process. When the missing responses were imputed as previously described except for the cases of PCR-detected new infections where the missing observations were imputed ACPR, the analyses using the Gibbs sampler failed to converge.

From the clinical standpoint, it is worth noting that the present data set concerned the use of highly efficacious anti-malarial combination drugs, thus explaining the absence of the ETF category. AMLM is already an alternative to ASAQ in Cameroon. Both ASAQ and AMLM treatments are recommended by the WHO, based on several published trials, comparing different subsets of the treatments listed in the present analysis [28-38]. The results of the present analysis complete the previous meta-analysis based on a binary outcome at a fixed time point, where it was concluded that AMLM appeared to be the most effective drug with no treatment failure due to recrudescence, closely followed by DHPP. However, the previous analysis did not take into account the individual repeated measurements.

**Table 5:** Sensitivity analysis: Estimated effects in the PP data set and the imputed data sets

	**PP dataset**			**Imputed dataset**		
**Covariates**	**OR**	**95% CI**		**OR**	**95% CI**	
Study 3-1	1.11	0.16	11.58	3.72	0.70	19.68
Study 4-1	2.17	0.29	16.52	2.06	0.43	9.92
Study 5-1	0.51	0.07	3.857	2.23	0.05	9.03
AQ	1.47	0.24	8.89	1.84	0.39	8.64
AMLM	3.84	0.47	31.35	5.40	0.88	33.31
ASCD	0.29	0.02	3.30	0.41	0.05	3.27
ASSP	4.88	0.76	31.20	3.95	0.80	16.61
DHPP	17.89*	4.70	67.99	15.64*	3.59	68.10
Day21-14	0.05*	0.01	0.21	0.02*	0.01	0.05
Day28-14	0.05*	0.01	0.41	0.02*	0.01	0.04

Model M3, study # 2 excluded. OR: Odds Ratio, 95% CI: confidence interval. *: significant at the 5% level.

DHPP showed a higher efficacy as compared to the reference treatment ASAQ in all tested models, whereas ASCD appeared less efficacious than ASAQ. AMLM did not differ significantly in efficacy from ASAQ. It should be remembered that the present analysis discarded study #2, as it was unconnected to others, increasing the power for comparison between the remaining treatment arms. The final result is in agreement with the meta-analysis conducted by Sinclair *et al.*[39], which was based on the binary outcome on Day 28. In that study, both DHPP and ASMQ appeared more efficacious than AMLM. ASMQ, which is not recommended by the WHO in Africa at present, although it is the first-line treatment in Southeast Asia, was not connected to the other treatment arms in the analysed network of randomised trials, and could only be compared to AQSP, showing a significantly higher efficacy. Treatment failure may have several origins including individual pharmacokinetic and pharmacodynamic variations and intensity of transmission. For instance, as CD has a shorter half-life than the other drugs, new infections could occur more easily than with other drugs, which explains why in a non PCR-corrected data analysis ASCD appears less effective. While a 100% full success rate (ACPR) represents the optimal target when treating acute malaria, it is worth noting that incorporating the information about the other intermediary states and the absence of parasitaemia appear to be of importance, at least from the public health standpoint to limit the burden of circulating parasites. Taking into account these intermediary outcomes could more adequately participate in the evaluation of different public health policies for malaria control in parallel to other validated interventions, such as the distribution of insecticide-impregnated bednets, environmental drainage and other mosquito control measures.

## Abbreviations

ACT, Artemisinin-based combination therapy; AMLM, Artemether - lumefantrine; ASAQ, Artesunate - amodiaquine; ASCD, Artesunate - chlorproguanil - dapsone; ASSP, Artesunate-sulphadoxine-pyrimethamine; DHPP, Dihydroartemisinin - piperaquine; ASMQ, Artesunate - mefloquine; AQSP, Amodiaquine - sulphadoxine - pyrimethamine; PP, Per protocol; WHO, World Health Organization.

## Competing interests

The authors declare that they have no competing interests.

## Authors’ contributions

SWY developed the analysis plan and carried out both the statistical analyses and software implementations under the close supervision of AS and JCT. She also drafted the manuscript. LKB was responsible for the overall data collection and supervision of clinical trials. All authors read and approved the final manuscript.
